# Knowledge and Awareness of Systemic Lupus Erythematosus Among the Population of Jeddah, Saudi Arabia: A Cross-Sectional Study

**DOI:** 10.7759/cureus.51929

**Published:** 2024-01-09

**Authors:** Abdullah M Alagha, Sami M Bahlas, Abdulaziz A Alariefy, Saher F Alqarni, Faisal A Almahyawi, Saleh A Alariefy

**Affiliations:** 1 Faculty of Medicine, King Abdulaziz University, Jeddah, SAU; 2 Rheumatology Division, Internal Medicine, King Abdulaziz University, Jeddah, SAU

**Keywords:** internal medicine and rheumatology, cross sectional studies, jeddah city, sle, autoimmune diseases

## Abstract

Background

The immune system, composed of various molecules and cells, protects humans from cancer and pathogens. A breach of tolerance, known as autoimmune disease (AD), is the root of these diseases. Systemic lupus erythematosus (SLE) is an autoimmune condition characterized by chronic inflammation, causing tissue damage in various organ systems. The disease is influenced by hormonal, environmental, and genetic factors. The pathophysiology is unclear, and 20% to 30% of patients have a persistent illness. SLE affects young females more than males, and treatments focus on organ manifestations. Despite advancements and better diagnoses, SLE continues to contribute significantly to morbidity and early mortality.

Objective

This study aims to assess knowledge of SLE among the general population of Jeddah, Saudi Arabia.

Methodology

An online cross-sectional survey using Google Forms was conducted for Jeddah residents aged 18 and above. The survey was open for responses from August 2023 to October 2023.

Results

The study included 479 participants, with 19 (25%) males and 57 (75%) females diagnosed with SLE. The majority of these individuals were housewives and unemployed. The majority were married (46, 60.5%), with only 25 (32.9%) being single. Among healthy participants, there were 173 (42.9%) males and 230 (57.1%) females, with a majority being housewives and government employees (95, 23.6%). Singles accounted for 124 (30.8%), while married individuals constituted 253 (62.8%). Among the healthy population respondents, 254 (63%) lacked knowledge about SLE treatment, while 40 (52.6%) SLE patients believed that a combination of chemotherapy, malaria medication, and steroids was the best treatment. The study found that 393 (82%) of the sample had heard about SLE, and 250 (52%) believed it was not a contagious disease. More than 30 were unaware of SLE. The majority of the respondents felt they needed more awareness and health promotion about SLE, with 410 (85.77%) stating they needed more promotion. The majority of the people believed SLE was dangerous to some extent.

Conclusions

This study revealed the need and necessity of awareness of SLE among the general community of Jeddah. We advocate undertaking disease awareness programs and activities to increase general community knowledge and awareness of SLE in the city of Jeddah.

## Introduction

The body's immune system is composed of a vast array of molecules and cells that have evolved to protect humans against cancer and invading pathogenic microbes [1]. Failure to discriminate self from non-self can be referred to as a breach of tolerance and is the root of an autoimmune disease (AD) [2]. Although the pathophysiology and etiology of ADs are not completely known, genetic, environmental, and their interactions play a significant role in the development of these diseases [3]. Systemic lupus erythematosus (SLE) is an autoimmune condition characterized by severe and chronic inflammation, resulting in tissue damage across multiple organ systems, with the renal system commonly affected [4]. The condition can impact all other systems as well. This makes SLE a heterogeneous disease with variable presentations [5]. SLE is hypothesized to result from a confluence of hormonal, environmental, and genetic variables. The pathophysiology of SLE, however, remains unclear and complicated [6]. Anti-dsDNA and anti-Sm, which are highly specific for SLE, play a key role in immune complex formation and disease progression [7]. The majority of patients with SLE have a waxing and waning disease history, and this adds to the difficulty in monitoring the disease and deciding the therapeutic options, whereas 20% to 30% of patients with SLE have a persistently active or quiescent illness [8]. It was estimated that 19.28 per 100,000 people in central Saudi Arabia had SLE [9]. SLE is known to affect young females nine times more than males [4]. To achieve minimal disease activity, treatments are focused on the particular organ manifestation. Now, we have relevant definitions for clinical remission (CR) and low lupus disease activity state (LLDAS), and we should apply the treat-to-target (T2T) approach in disease management [10]. Despite several therapeutic advancements and improved diagnoses, SLE still contributes significantly to morbidity and early mortality [11].

In Al-Riyadh, a study published in 2019 found that the majority of the participants had low awareness and poor knowledge about SLE [12]. Another study conducted in Makkah demonstrated that there was an insufficient level of knowledge and awareness of SLE among the study population [13]. Furthermore, research carried out in Al-Dammam City found that the general population was unaware of SLE and had some misconceptions about it [14]. A survey done in Al-Ahsa City in 2018 showed that students had minimal understanding of the SLE and some misconceptions regarding it [15]. In two studies done by Abha and Al-taif, participants' awareness of SLE was weak, and there were many misconceptions regarding important SLE facts [16-17]. In India, a survey conducted in 2017 to gauge community knowledge and awareness of SLE revealed that the majority of participants knew little about the illness as an unusual disease affecting the general population [18].

To the best of our knowledge, no prior research of a similar nature has been undertaken in the city of Jeddah. Thus, our study aimed to investigate knowledge and awareness of SLE among the general population of Jeddah, Saudi Arabia.

## Materials and methods

Study design and settings

A cross-sectional study was conducted in Jeddah, Saudi Arabia, from August 2023 to October 2023, to assess the knowledge and awareness of SLE among the population. OpenEpi version 3.01 was used to calculate the minimum required sample size. A sample size of 384 individuals was deemed the smallest acceptable to maintain a 95% confidence interval [19].

Ethical considerations and study participants

A total of 479 citizens from Jeddah participated in the present investigation. Both male and female residents of Jeddah, Saudi Arabia, who were at least 18 years old and had given their consent to participate, were eligible to take part. Participants who were outside Jeddah or less than 18 years old were excluded. The study was approved by the King Abdulaziz University Ethics Committee (Reference no. 449-23).

Data collection

We used a validated assessment tool that was taken from a previously published study [13]. Participants were approached through social media platforms with a link to the study questionnaire, which was available in both Arabic and English. The questionnaire included a consent form for the use of participant data in research. According to the requirements of the university ethics committee for cross-sectional studies, each participant provided informed consent. The questionnaire comprised two sections. The first section aimed to collect sociodemographic information, while the second section included questions related to knowledge and awareness of SLE.

Data analysis

For qualitative variables, frequency tables were computed to illustrate demographic variables, including gender, occupation, education, marital status, and monthly income. For quantitative variables, measures of central tendency and dispersion were used to describe the data.

The Chi-square (X²) test was used to compare two groups of SLE awareness.

All data were stored and sorted using Microsoft Excel Version 2023, and the analysis was conducted using the IBM SPSS Statistics for Windows, Version 29 (IBM Corp., Armonk, NY). A significance level of less than 0.05 was considered statistically significant.

## Results

A total of 593 responses were collected from the electronic questionnaire. After excluding 114 subjects who did not meet the inclusion criteria, 479 subjects were successfully enrolled in the study (*N* = 479, mean = 39.95, 95% confidence interval [CI] 38.78-41.09, standard deviation [SD] = 13.41, 95% CI 12.56-14.32).

Demographics of the participants

Among the study participants who were diagnosed with SLE, there were 19 (25%; *P*-value = 0.0001) males and 57 (75%; *P*-value = 0.0001) females, with the majority of them being housewives (19, 25%) and unemployed (18, 23.7%). Also, 25 (32.9%) subjects were single and 46 (60.5%) were married.

**Figure 1:**
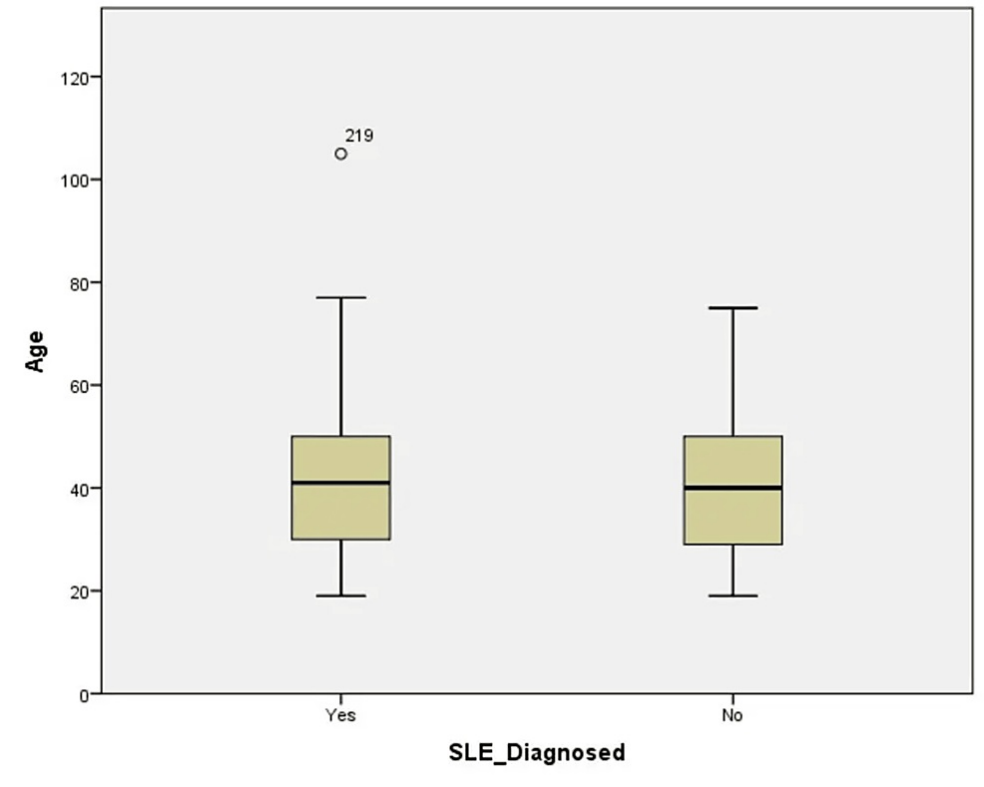
Boxplot illustrating the distribution of subject demographics based on age and SLE diagnosis. SLE, systemic lupus erythematosus

On the contrary, among healthy participants, there were 173 (42.9%; *P*-value = 0.0001) males and 230 (57.1%; *P*-value = 0.0001) females; the majority of them were housewives (83, 20.6%) and government employees (95, 23.6%). In addition, 124 (30.8%) subjects were single and 256 (62.8%) were married. Other demographic parameters were given in Table 1 and Figure 1.

**Table 1:** Demographic characteristics of the participants.

		SLE diagnosed?			
		Yes		No	
		N	%	N	%
Gender	Female	57	75.0	230	57.1
	Male	19	25.0	173	42.9
Occupation	Student	10	13.2	71	17.6
	Housewife	19	25.0	83	20.6
	Government employee	15	19.7	95	23.6
	Nongovernment employee	14	18.4	77	19.1
	Unemployed	18	23.7	77	19.1
	Retired	0	0.0	0	0.0
Education	Primary school	0	0.0	0	0.0
	Middle school	2	2.6	10	2.5
	High school	19	25.0	62	15.4
	College	24	31.6	162	40.2
	Graduated	19	25.0	105	26.1
	Post-graduation studies	10	13.2	63	15.6
	Can read and write	1	1.3	1	0.2
	Nonliterate	1	1.3	0	0.0
Marital status	Single	25	32.9	124	30.8
	Married	46	60.5	253	62.8
	Divorced	3	3.9	22	5.5
	Widowed	2	2.6	4	1.0
Monthly income	Less than 5,000	27	42.9	153	42.9
	5,000-10,000	22	34.9	91	25.5
	10,000-20,000	14	22.2	113	31.7

Questionnaire responses among participants

Regarding the treatment option question among the healthy population, the majority of respondents did not know about the treatment for SLE (*N* = 254, 63%). In contrast, SLE subjects responded that a combination of chemotherapy, malaria medication, and steroids was the treatment for SLE patients (*N* = 40, 52.6%), as described in Table 2.

**Table 2:** Subject responses from the electronic questionnaire about SLE treatment options. SLE, systemic lupus erythematosus

	SLE patients		Healthy subjects	
	N	%	N	%
Chemotherapy	2	2.6	15	3.7
Steroids	16	21.1	52	12.9
Malaria medications	10	13.2	5	1.2
Combination of the above medications	40	52.6	77	19.1
I don’t know	8	10.5	254	63

SLE level of awareness

**Figure 2:**
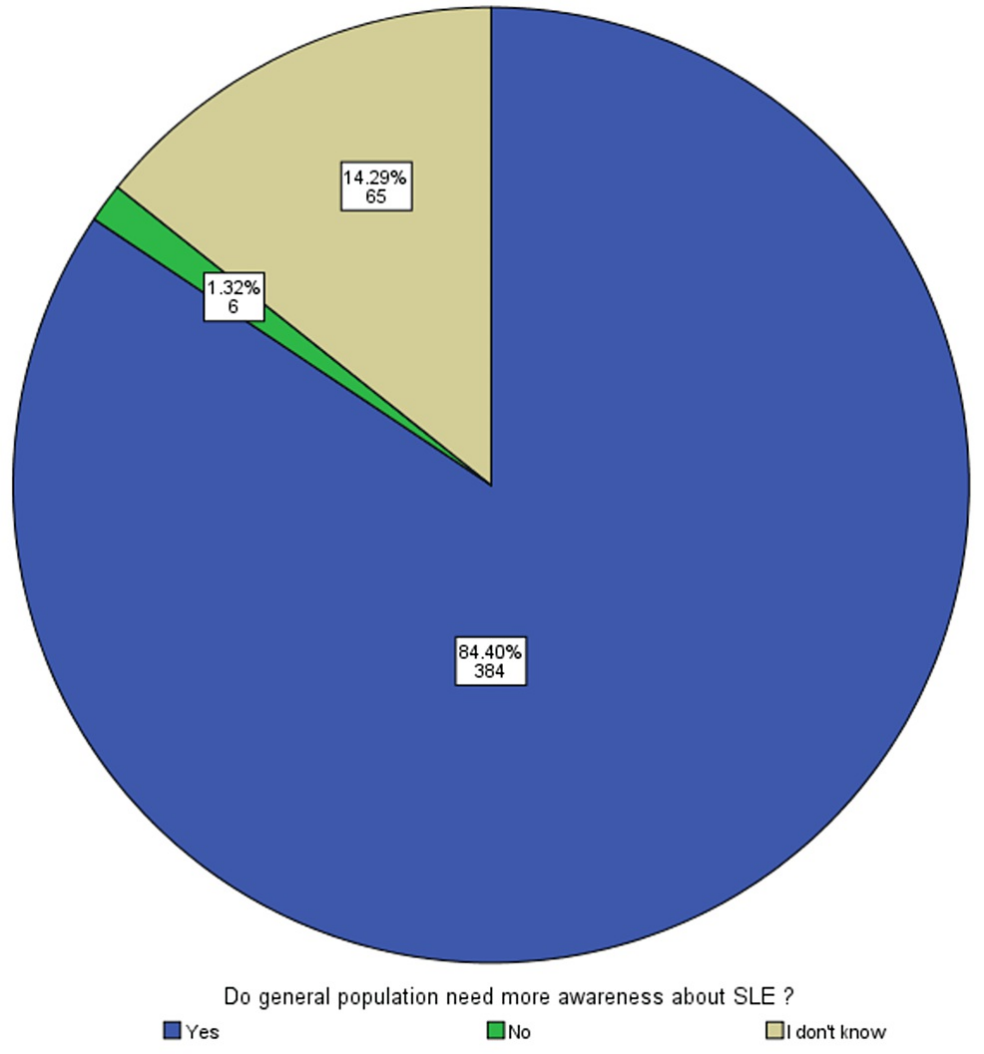
The pie graph showing general population awareness and lack knowledge about SLE. SLE, systemic lupus erythematosus

A total of 393 (82%) individuals in the sample heard about SLE, with 250 (52%) believing that SLE was not a contagious disease, while only 13 (3%) thought it was contagious. Additionally, most of the population thought that SLE is a dangerous disease to some extent, as given in Table 3. Moreover, many people above the age of 30 years old were unaware of SLE (age group: 31-39, *N *= 81, 87.1%; 40+, *N *= 226, 89.3%; *P*-value < 0.001) compared with subjects who were aware (age group: 31-39, *N *= 12, 12.9%; 40+, *N *= 27, 10.7%; *P*-value < 0.001). Furthermore, males tend to be more knowledgeable about SLE (unaware: *N *= 137, 68.5%; aware, *N *= 63, 31.5%) than female subjects (unaware: *N *= 266, 90.8%; aware, *N *= 27, 9.2%; *P*-value < 0.001; *P*-value < 0.001), as shown in Table 4. Also, when subjects were asked whether they needed more awareness and health promotion about SLE, most of them responded that they need more promotion (*N* = 410, 85.77%), while only a minority stated that they do not need promotion (*N* = 5, 1.05%), as shown in Figure 2.

**Table 3:** Subjects responses from the electronic questionnaire about SLE awareness.

		Number of responses, *N*	%
Have you ever heard of the term systemic lupus erythematosus (SLE)?	Yes	393	82
	No	85	18
Is SLE contagious?	Yes	13	3
	No	250	52
	I don't know	215	45
Is SLE dangerous?	Very dangerous	54	11
	Dangerous	99	21
	Moderately dangerous	308	64
	Small chance of causing danger	14	3
	I don’t know	3	1
Does SLE affect females only?	Yes	185	39
	No	24	5
	I don’t know	269	56
Can SLE be diagnosed with a single blood test?	Yes	61	13
	No	113	24
	I don’t know	304	64
Is SLE treatable?	Yes	197	41
	No	103	22
	I don’t know	178	37
Is SLE preventable?	Yes	106	22
	No	98	21
	I don’t know	274	57
Does SLE have complications?	Yes	36	8
	No	212	44
	I don’t know	230	48
What are SLE medications?	Chemotherapy	17	4
	Steroids	68	14
	Malaria medications	15	3
	Combination of the above medications	116	24
	I don’t know	262	55
Does SLE affect fertility?	Yes	84	18
	No	52	11
	I don’t know	342	72
Does SLE cause fetal anomalies?	Yes	141	29
	No	26	5
	I don’t know	311	65

**Table 4:** Relationship between SLE awareness of subjects and their demographic characteristics. SLE, systemic lupus erythematosus

		SLE awareness				*P*-value
		Unaware		Aware		
		N	%	N	%	<0.001
Age groups (years)	18-20	32	72.7	12	27.3	
	21-25	37	56.1	29	43.9	
	26-30	27	73.0	10	27	
	31-39	81	87.1	12	12.9	
	40+	226	89.3	27	10.7	
Gender	Female	266	90.8	27	9.2	<0.001
	Male	137	68.5	63	31.5	
Occupation	Student	60	63.2	35	36.8	<0.001
	Housewife	88	86.3	14	13.7	
	Government employee	102	92.7	8	7.3	
	Nongovernment employee	71	78	20	22	
	Unemployed	82	86.3	13	13.7	
	Retired	0	0.0	0	0.0	
Education	Primary school	0	0.0	0	0.0	<0.001
	Middle school	9	75	3	25	
	High school	66	77.6	19	22.4	
	College	147	75	49	25	
	Graduated	111	89.5	13	10.5	
	Post-graduation studies	67	91.8	6	8.2	
	Can read and write	2	100	0	0.0	
	Nonliterate	1	100	0	0.0	
Marital status	Single	117	71.8	46	28.2	<0.001
	Married	259	86.6	40	13.4	
	Divorced	22	88	3	12	
	Widowed	5	83.3	1	16.7	
Monthly income	Less than 5,000	141	73.1	52	26.9	<0.001
	5,000-10,000	93	81.6	21	18.4	
	10,000-20,000	117	92.1	10	7.9	
SLE acquisition	Relatives	90	98.9	1	1.1	<0.001
	Colleague	67	97.1	2	2.9	
	No one	201	70.3	85	29.7	
	Others	45	95.7	2	4.3	

## Discussion

SLE, sometimes known as lupus, is a complex autoimmune disease that affects multiple organs with a wide range of different presentations that can result in mild to life-threatening illness. Patients with lupus continue to face a significant risk of morbidity and mortality despite recent advances in early diagnosis, treatments, and T2T approaches (low disease activity). For example, approximately 10% of patients with lupus nephritis (LN) develop end-stage kidney disease [20]. Because of this, the purpose of our study was to evaluate the degree of lupus awareness and knowledge in Jeddah, Saudi Arabia.

A total of 479 participants met the inclusion criteria for our study, and 76 of them were diagnosed with SLE. Fifty-seven (75%) of the participants with an SLE diagnosis were female, while 19 (25%) were male. Among the 403 people without an SLE diagnosis, 230 (57.1%) were female and 173 (42.9%) were male. In a study conducted in Makkah, Saudi Arabia, 54.5% of the participants were female and 45.5% were male [13].

We investigated the awareness levels regarding the term SLE among the participants. The results revealed a relatively high level of awareness, with 393 (82%) respondents indicating that they had heard of SLE, while 85 (18%) reported that they had not heard of it. Interestingly, these findings closely align with the results of a similar study conducted in Al-taif, where 81.1% had heard of SLE and 18.9% hadn't [17]. However, when comparing our study with research conducted in Makkah, we observed a significantly different scenario. In Makkah, only 52.3% of the participants had heard of SLE, while a substantial 47.7% remained unaware of the term [13]. It is noteworthy that while 393 (82%) participants in our study had heard of the term SLE, their level of understanding regarding the complications associated with this autoimmune condition appeared to be comparatively lower. Among the participants, only 36 (8%) reported that they knew about the complications associated with the disease, while 212 (44%) confidently stated that they believed SLE did not have complications. Strikingly, a significant 230 (48%) respondents admitted that they did not know whether SLE had associated complications or not. These findings suggest that there may be a gap in knowledge not only concerning the existence of SLE but also regarding its complications and potential health implications.

We explored the relationship between awareness of SLE and demographic characteristics. Surprisingly, the data revealed that males displayed a higher level of awareness compared to females (*P *< 0.001). This goes with findings from a study conducted in Abha, where males were also found to be more aware of SLE than females (*P *= 0.018) [16]. In contrast, a study conducted in Makkah demonstrated an inverse trend, revealing that females were more aware of SLE with a statistically significant difference (*P *= 0.001) [13]. In Riyadh, on the other hand, no significant difference in SLE awareness between males and females was observed (*P *= 0.304) [12]. These variations highlight the influence of regional and cultural factors on public awareness, emphasizing the need for tailored educational strategies that account for local demographics in each region to effectively disseminate knowledge about SLE.

Our research indicates that students exhibit a significantly higher level of awareness regarding SLE when compared to housewives, government employees, nongovernment employees, and the unemployed, with a statistically significant difference in awareness levels (*P *< 0.001). These findings align with similar trends observed in studies conducted in Makkah and Abha, which consistently identified students as having the highest level of awareness of SLE [13,16]. The disparity in awareness could be attributed to several factors. Students are often actively engaged in educational environments where health topics are discussed, making them more likely to encounter information about SLE. Additionally, educational institutes tend to include teachings about SLE as part of their curriculum, contributing to increased awareness among students. In contrast, employees and nonemployees may have limited exposure to health-related educational resources due to their daily work routines and may not have the same level of access to healthcare information.

Finally, we found that people who did not know anyone with SLE had a much higher awareness level compared to those who did know someone with SLE, with a significant difference (*P *< 0.001). This was unexpected given that individuals who know someone with SLE should be more aware. This finding shares a similar result from a study conducted in Riyadh, where those who did not know anyone with SLE also had higher awareness [12]. However, this contrasts with the study in Makkah, which found that knowing someone diagnosed with SLE was linked to higher awareness [13].

Limitations

Because this study was limited to the people of Jeddah, generalizing the results may be difficult. Furthermore, because the information was gathered using an online survey, the study was limited to a sample of the population with Internet access. There might also be recall bias.

## Conclusions

This study indicated that the participant's knowledge about SLE was inadequate and deficient, and there are some misconceptions about crucial information about the disease. Increasing awareness levels among citizens in Jeddah will be useful. Therefore, public awareness campaigns, social media, social media influencers, and e-brochures are needed to assist society in becoming aware of the disease's clinical symptoms and signs, severity, consequences, and therapy. Finally, we recommend performing more research in various locations in Saudi Arabia to find out the kingdom's degree of knowledge and awareness of SLE.
